# Silver Nanoparticles in Zebrafish (*Danio rerio*) Embryos: Uptake, Growth and Molecular Responses

**DOI:** 10.3390/ijms21051876

**Published:** 2020-03-09

**Authors:** Liyuan Qiang, Zeinab H. Arabeyyat, Qi Xin, Vesselin N. Paunov, Imogen J. F. Dale, Richard I. Lloyd Mills, Jeanette M. Rotchell, Jinping Cheng

**Affiliations:** 1 State Key Laboratory of Estuarine and Coastal Research, East China Normal University, Shanghai 200062, China; qiangziaiyuan@163.com (L.Q.); xinxin8922@126.com (Q.X.); 2 Department of Marine Biology, the University of Jordan, Aqaba branch, Aqaba 77111, Jordan; z.arabeyyat@ju.edu.jo; 3 Department of Chemistry and Biochemistry, University of Hull, Cottingham Road, Hull HU6 7RX, UK; V.N.Paunov@hull.ac.uk; 4 School of Biological, Biomedical, and Environmental Sciences, University of Hull, Cottingham Road, Hull HU6 7RX, UK; imogen.dale@hotmail.com (I.J.F.D.); rilloydmills1@sheffield.ac.uk (R.I.L.M.); 5 Hong Kong Branch of Southern Marine Science and Engineering Guangdong Lab (Guangzhou) & Department of Ocean Science, School of Science, the Hong Kong University of Science and Technology, Kowloon, Hong Kong

**Keywords:** silver nanoparticles, molecular response, continuous exposure, uptake, developmental toxicity, embryos (*D. rerio*)

## Abstract

Silver nanoparticles (AgNPs) are widely used in commercial applications as antimicrobial agents, but there have recently been increasing concerns raised about their possible environmental and health impacts. In this study, zebrafish embryos were exposed to two sizes of AgNP, 4 and 10 nm, through a continuous exposure from 4 to 96 h post-fertilisation (hpf), to study their uptake, impact and molecular defense responses. Results showed that zebrafish embryos were significantly impacted by 72 hpf when continuously exposed to 4 nm AgNPs. At concentrations above 0.963 mg/L, significant in vivo uptake and delayed yolk sac absorption was evident; at 1.925 mg/L, significantly reduced body length was recorded compared to control embryos. Additionally, 4 nm AgNP treatment at the same concentration resulted in significantly upregulated *hypoxia inducible factor* 4 (*HIF4*) and *peroxisomal membrane protein 2* (*Pxmp2*) mRNA expression in exposed embryos 96 hpf. In contrast, no significant differences in terms of larvae body length, yolk sac absorption or gene expression levels were observed following exposure to 10 nm AgNPs. These results demonstrated that S4 AgNPs are available for uptake, inducing developmental (measured as body length and yolk sac area) and transcriptional (specifically *HIF4* and *Pxmp2*) perturbations in developing embryos. This study suggests the importance of particle size as one possible factor in determining the developmental toxicity of AgNPs in fish embryos.

## 1. Introduction

Nanomaterials have been defined as materials with at least one dimension smaller than 100 nm [1,2]. These materials include produced nanomaterials, such as diesel exhaust materials or airborne combustion byproducts, as well as nanosized materials which naturally occur in the environment, such as viruses or volcanic ash [1,2]. Silver nanoparticles (AgNPs) are widely used nanomaterials in commercial products, with uses ranging from disinfecting medical products to household items and water treatment [3]. In 2015, over 410 products on the global market contained AgNPs, with an annual global production of > 550 t [4,5]. It is projected that the nanotechnology industry will continue to grow significantly, and the specific production of AgNPs is expected to reach approximately 800 t by 2025 [6,7]. Each year, approximately 63 t of AgNPs enter water bodies worldwide [8], leading to predicted environmental concentrations of ~10–1800 ng/L and 40–80,000 µg/kg in surface waters and sediments, respectively, by 2020 [9]. The potential impacts of increased AgNP concentrations on aquatic organisms are not fully understood yet.

Exposure to AgNPs has various negative impacts on fish. Several studies have focused on biological uptake and whether such AgNPs can cross fish embryo chorionic membranes [10,11,12]. Previous studies have reported that metal nanoparticles can pass through chorionic pores but do so randomly, resulting in unpredictable effects [12]. Gao et al. [10] also observed uptake within the embryo cardiovascular system following exposure to 1000 µg/L AgNPs (size range 50 nm) at 24 to 96 h post-fertilisation (hpf), as well as later development abnormalities [11]. A similar exposure regime of 3–1000 µg/mL AgNPs (size range 16–58 nm) also resulted in later development abnormalities in early medaka embryos, including chorionic membrane disruption [13]. Oxidative stress (at 0.05–120 mg/L AgNPs), respiratory toxicity (at > 72 mg/L AgNPs) and locomotion disorders (at > 0.47 mg/L AgNPs) have also been reported following controlled AgNP exposure in fish [13,14,15,16,17].

In terms of the mechanisms of AgNPs’ toxicity, several studies have suggested that the toxicity of AgNPs is associated with the uptake of silver in the exposed organisms [18,19], yet others have demonstrated that particulate effects are responsible [20,21]. Regarding uptake, Xin et al. [22] reported an accumulation of AgNPs in the head and trunk region of exposed zebrafish embryos under a static exposure with the AgNP exposure media replaced every 24 h. Similarly, Osborne et al. [23] reported silver content in the gills and intestine of exposed adult zebrafish following an exposure to 20–110 nm size range AgNPs for 4 days, wherein the exposure media was refreshed every third day. Additionally, Griffitt et al. [21] detected silver in the gills and carcasses of adult female zebrafish exposed to 26.6 ± 8.8 nm size range AgNPs for 48 h. However, major factors contributing to the uptake and toxicity of AgNPs in fish embryos remain unclear. 

Furthermore, studies using fish have shown that silver ions alone, resulting from silver nanomaterial destabilisation, can cause oxidative stress in embryos [24] and adults [14], interfere with gill ion pumps in adults [25], increase mortality and morphological malformations in embryos (120 hpf at a dose of 3–100 nM) [26] and impair swimming behaviour and locomotion [15,17]. 

In the present study, the uptake, developmental impacts and associated molecular-level responses of early zebrafish embryos to continuous exposure to two sizes of AgNPs (4 and 10 nm) for 4 days without media replacement were investigated. A specific molecular-level approach was used, involving targeted gene transcripts *superoxide dismutase 2* (*SOD2*), *catalase (CAT)*, *hypoxia inducible factor* 4 (*HIF4*), *peroxisomal membrane protein 2 (Pxmp2)* and *mucosal secretion protein* (*Muc*), which are representative of oxidative stress and membrane functions. 

## 2. Results

### 2.1. Characterisation of AgNPs

**Figure 1:**
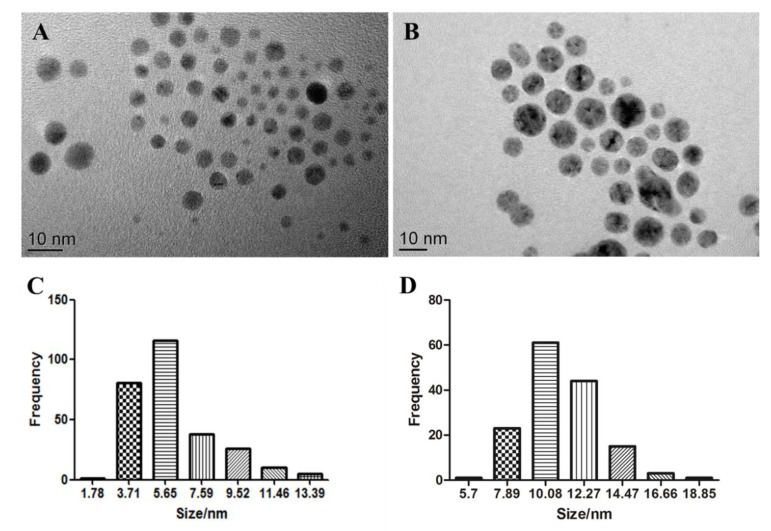
Transmission electron microscopy images of S4 (**A**) and S10 (**B**) silver nanoparticles (AgNPs). Histograms of the size distribution for S4 (**C**) and S10 (**D**).

The S4 AgNPs used in the study consisted of spherical shapes under TEM observation, with an average diameter of 5.14 ± 2.17 nm (Figure 1A). The size distribution histograms (Figure 1C) reveal that the size of S4 AgNPs ranged mainly from 3.7 to 5.7 nm. The S10 AgNPs used also consisted of spherical shapes under TEM observation, with an average diameter of 9.89 ± 2.03 nm (Figure 1B). The size distribution histograms (Figure 1D) reveal that the size of S10 AgNPs mainly ranged from 7.9 to 14.5 nm. TEM images also indicate that the AgNPs had a homogeneous dispersion in the aqueous solution (Figure 1A,B). 

### 2.2. Embryo Body Length and Yolk Sac Area Impacts

**Figure 2:**
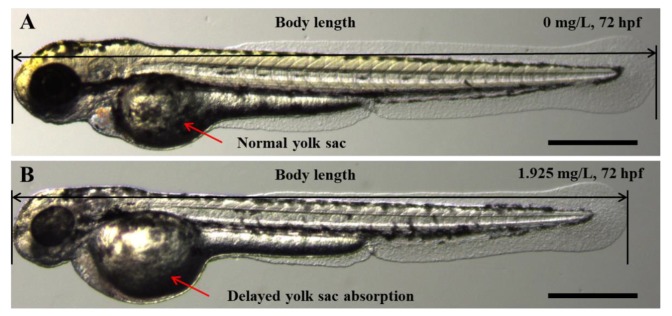
Representative phenotypes of zebrafish embryos upon exposure to AgNP concentration of (**B**) 1.925 mg/L, compared with the control (**A**) at 72 hpf. Scale bar = 500 μm.

**Figure 3:**
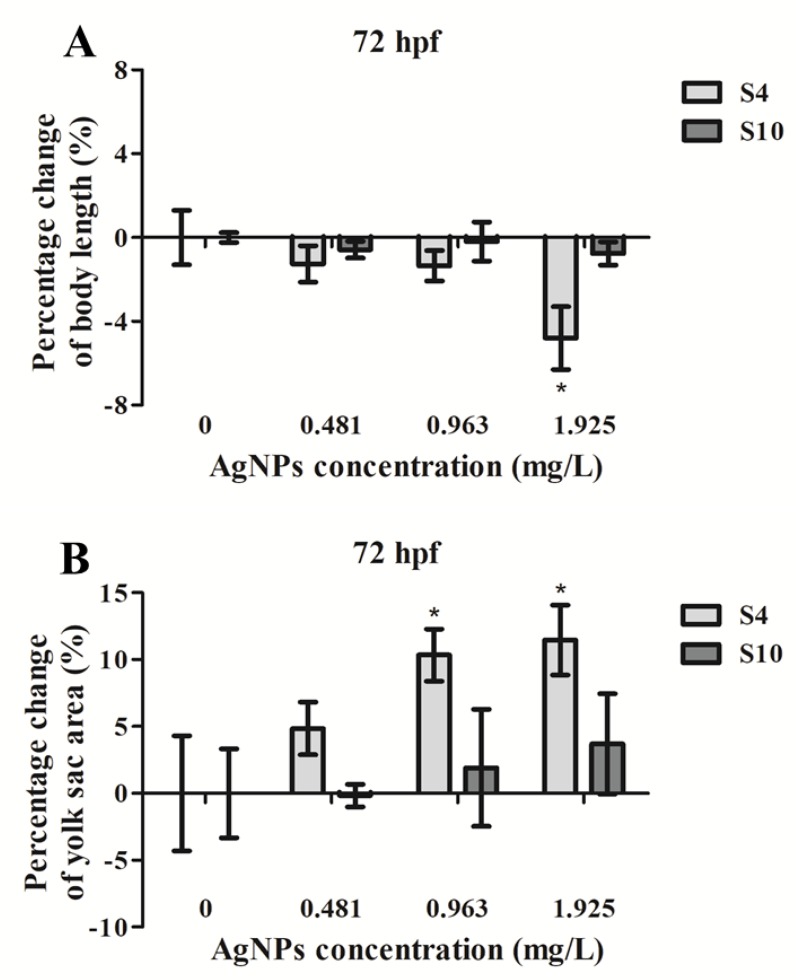
Percentage change of (**A**) body length and (**B**) yolk sac area of zebrafish embryos following exposure to AgNPs (at 0.481, 0.963, 1.925 mg/L) compared with the control (without AgNPs) at 72 hpf. Values represent mean ± standard error. Results have been normalised on control (0 µg/L). The body length of control embryos was (3.618 ± 0.047) mm (S4) and (3.603± 0.009) mm (S10), and the yolk sac area of control embryos was (0.328 ± 0.014) mm^2^ (S4) and (0.333 ± 0.011) mm^2^ (S10). * significant difference between samples and the control groups using one-way ANOVA. * *p* < 0.05. *n* = 4.

Exposure to S4 AgNPs disturbed the development of zebrafish larvae, including decreased body length (Figure 2A) and delayed yolk sac absorption (Figure 2B). Mean body length of zebrafish embryos at 72 hpf was significantly decreased when exposed to the highest dose (1.925 mg/L) of S4 AgNPs treatment compared to control embryos (Figure 3A). No significant differences in body length at the lower dose levels of S4 AgNPs were observed (Figure 3A). Similarly, yolk sac area (mm^2^) was significantly increased when exposed to the two highest doses (0.963 and 1.925 mg/L) of S4 AgNPs treatment compared to control embryos (Figure 3B). No significant differences in yolk sac area at the lower S4 AgNP dose levels were observed (Figure 3B). There was a trend of the yolk sac area of zebrafish larvae in the S4 AgNPs treatment groups to increase with increasing concentrations of AgNPs. Exposure to S10 AgNPs had no significant observable effects on body length or yolk sac area at all studied concentrations.

### 2.3. AgNP Uptake

**Figure 4:**
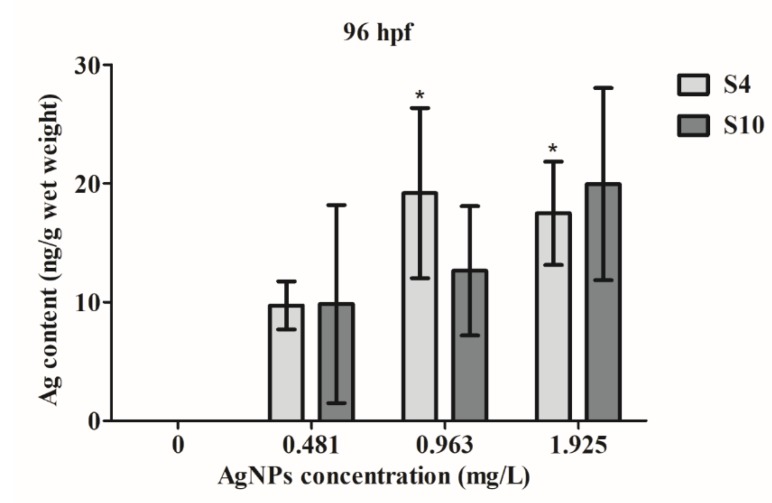
Silver content in wet weight of fish embryos exposed to AgNPs from 4 to 96 hpf. Values represent mean ± standard error. * significant difference between samples and the control groups using one-way ANOVA. * *p* < 0.05. *n* = 4.

ICP-MS analysis revealed that zebrafish embryos took up AgNPs (Figure 4), with significant uptake at higher concentrations of S4 AgNPs over 0.963 mg/L. Even at a low concentration of 0.481 mg/L, there was an increased uptake of silver content in the exposed fish embryos, although the amount was not significant. Silver content did not significantly increase following exposure to any concentration of the S10 size range AgNPs (Figure 4). Similarly, however, an increased silver content level was observed in the S10 AgNP treatment groups (Figure 4) as compared to the blank control. The silver content in fish embryos reached around 20 ng/g wet weight at higher dose treatments.

### 2.4. qPCR Analysis of Candidate Gene Expression in Fish Embryos

**Figure 5:**
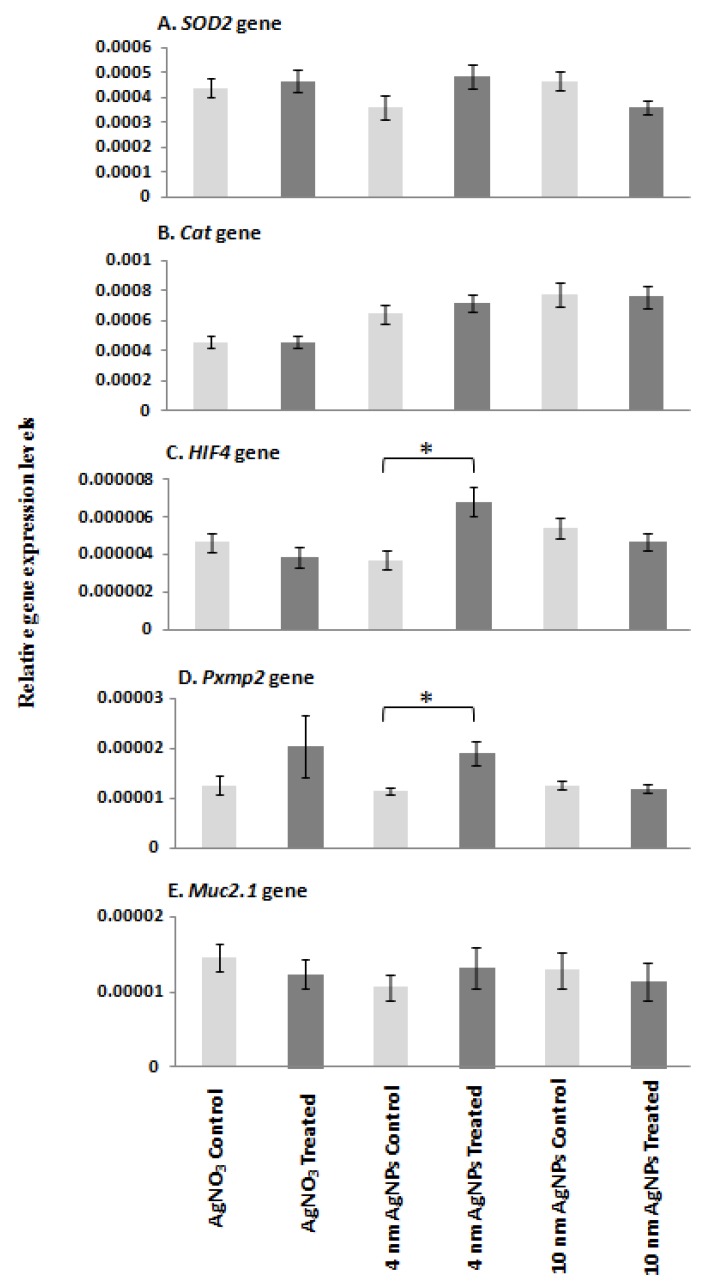
qPCR mRNA transcript levels for (**A**) *SOD2*, (**B**) *CAT*, (**C**) *HIF4*, (**D**) *Pxmp2* and (**E**) *Muc* genes in zebrafish embryos at 96 hpf following exposure to AgNO_3_ (18 µg/L Ag+), S4 AgNPs (1.925 mg/L), and S10 AgNPs (1.925 mg/L). Values represent mean ± standard error. * significant difference between samples and the control groups using either two-sample t-test or Mann–Whitney test. * *p* < 0.05. *n* = 4.

The expression level of each target mRNA was analysed in controls, silver-ion-treated and S4- and S10-AgNP-treated (1.925 mg/L) *D. rerio* embryos using qPCR (Figure 5). *HIF4* and *Pxmp2* mRNA expression was significantly upregulated (*p* < 0.05) following treatment with 1.925 mg/L of S4 AgNPs relative to control embryos (Figure 5C,D). *SOD2*, *CAT* and *Muc* mRNA expression levels showed no significant changes in AgNP-treated embryos compared with corresponding control embryo samples (Figure 5A,B,E). No significant differences in any of the gene expression levels were observed following exposure to S10 size range AgNPs or silver ions alone (Figure 5A–E).

## 3. Discussion

For the first time, this study provided a link between AgNP exposure and uptake and perturbation of membrane protein mRNA expression, as well as previously reported *hypoxia inducible factor* gene expression, in fish. Specifically, we were the first to observe significantly increased expression of a membrane transporter protein, *Pxmp2*, and hypoxia inducible factor *HIF4* mRNA expression in zebrafish embryos exposed to small size range (4 nm) AgNPs. 

The size of the AgNPs may be one important factor, among many, in determining their availability in fish embryos. This study provided evidence for significantly enhanced uptake and developmental toxicity of smaller sized AgNPs with a diameter of 4 nm, as compared to that of 10 nm AgNPs in zebrafish embryos. The ICP-MS data showed that exposure to S4 AgNPs from 4 to 96 hpf resulted in significantly enhanced silver uptake in zebrafish larvae, while no significant differences were observed in the silver uptake levels upon exposure to S10 AgNPs. Previous studies have shown that the size (and coating/suspension media) of AgNPs can determine the level of uptake [11,26,27], suggesting that the smaller size ranges up to 20 nm are more likely to be taken up than larger sized AgNPs. Bar-Ilan et al. [26] also reported that the biological impacts observed, including various morphological malformations and yolk sac oedema, significantly increased following exposure to 3 nm range AgNPs from 4–120 hpf. Correspondingly, our observations were that the body length was significantly reduced while yolk sac diameter was increased at the highest concentration of 1.925 mg/L for S4 AgNP exposure. In contrast, Muth-Kohne et al. [28] reported a decrease in egg yolk sac size following exposure to AgNPs (< 20 nm size) at 48 hpf, using a significantly higher dose of 73 mg/L. It is known that silver can accumulate in tissues both in the form of ions and in the form of nanoparticles [29]. The silver content measurement result in the present study could not ascertain the form of metal inside the tissue, which is one of the drawbacks of using ICP-MS to examine the uptake of nanometals. The overall uptake of silver content in zebrafish larvae in the present study was relatively low, which might have been due to the surface coating used in the studied AgNPs. Particle size and surface charge of nanoparticles were shown to have significant impacts on nanoparticles’ toxicity and interactions with biological membranes [30]. In the present study, both S4 and S10 AgNP exposure generated a low toxicity effect in the treated fish embryos, with a slightly enhanced toxic effect with S4 AgNPs. 

During the development of zebrafish (*Danio rerio*), baby fish hatch from around 48 hpf. Before hatching, the uptake of AgNPs in zebrafish embryos is affected by the chorion. The zebrafish embryo is enclosed by the chorion, which acts as a protective barrier against external toxic substances. The zebrafish chorion has pores of 0.5–0.7 µm diameter [11,31], which are necessary for oxygen and nutrient transportation for the embryos [32]. Arguably, the AgNPs entering the chorion represent an opportunity for uptake of AgNPs in the embryo tissues [12,18,33] or for later development abnormalities [11,13]. Compared to S10 AgNPs, S4 AgNPs with a smaller size have a larger surface area that can enhance their adherence to the embryo chorion, which may contribute to the developmental toxicity of AgNPs by blocking the chorion pores and thus obstruct the oxygen exchange.

After hatching, it is possible that both S4 and S10 AgNPs were taken up as food items and entered the body in that way, which might have contributed to the majority of uptake and toxicity of AgNPs. It has been reported that smaller particles have higher dissolution rates than larger particles due to their increased surface area [23]. As a result, the smaller sized AgNPs are more efficient carriers for local release and deposition of Ag ions in the fish body [11], which could result in site-specific disruption of Naþ/Kþ ATPase activity in the basolateral membranes [23]. As Naþ/Kþ ATPase activity has been reported to also be involved in the formation of tight junctions [34], disintegration of these adhesion sites could allow even more particle entry [35]. Osborne et al. [23] previously showed that silver content in the gills of adult zebrafish had significantly higher proportions of 20 nm silver particles than 110 nm particles after exposure for both 4 h and 4 days. 

In terms of molecular response, several studies have previously analysed various gene expressions in fish species in response to AgNP exposure, reporting significant changes in oxidative or general stress [13,14,16,27], toxicological [36,37] and morphological/neural development end-point markers [16,22]. *HIF4* mRNA expression was significantly elevated in S4-AgNP-treated embryos, which was indicative of a hypoxic cellular environment. Increased *HIF4* mRNA expression was consistent with another study that investigated medaka fish embryo lactate dehydrogenase (LDH) activity, which is regarded as a biomarker of hypoxic regulation in organisms/cells [13]. Wu and Zhou [13] also reported that medaka fish embryos exposed to AgNPs of size range 15–50 nm showed a dose-dependent (63–1000 µg/L) increase in LDH activity after three days of exposure. *HIF-1α* expression was also reported to be upregulated in adult fish (*P. promelas*) following exposure to 61.4 μg/L of poly (N-vinylpyrrolidone) AgNPs for 96 h [38]. It is thus evident, and consistent with the wider literature, that exposure to AgNPs at 1.925 mg/L resulted in a hypoxic and stressful environment within the embryos as measured at 96 hpf.

The increased expression of *Pxmp2* mRNA in embryos exposed to AgNPs was an important novel finding. This gene encodes a channel-forming protein that has so far only been characterised in the mammalian peroxisomal membrane, and is considered important in facilitating the continual flow of small solutes [39]. The Pxmp2 channel is estimated to have a diameter of 1.4 nm, with weak cation selectivity, and operates as a general diffusion pore in the membrane [39]. Both SOD and CAT enzymes are located within the peroxisome organelle, which scavenge for reactive oxygen species, specifically the oxygen (O_2_^.-^) free radical and hydrogen peroxide (H_2_O_2_), respectively, leading to the hydroxyl OH radical in the cell [40]. The increased *Pxmp*2 and *SOD* mRNA expressions may thus have been related to an increase in O_2_^.-^ levels within the AgNP-treated embryos at the early 96 hpf time point. Increased SOD activity following > 250 µg/L AgNP exposure has also been reported in medaka embryos up to 72 hpf, but was then reportedly followed by a decrease back to control values by the end of embryonic development [13]. In contrast, expression of *SOD1* mRNA in liver tissues of adult zebrafish and carp has been reported to be unaffected by treatment with significantly higher dose levels of 30, 60 and 120 mg/L [14] or lower dose levels of 25–200 μg/L of AgNPs after 48–96 h [41].

*Muc* mRNA expression levels in embryos were not altered as a result of either AgNP or silver ion exposure at the dose levels investigated. *Muc* is one of five *mucin* genes characterised in zebrafish, encoding a protein that forms a gel-based protective barrier in several tissues of adult fish, as well as in the head and tail region of a developing embryo, detectable from 48 hpf [42]. Studies using adult fish have previously investigated alterations in mucus secretion upon AgNP exposure [19], although no previous studies have focused on the mucus’ protective role in early-development embryos.

Any observed bioaccumulation of silver in embryos may have been due to both AgNPs and free silver ions. This could be part of the reason why previous studies have chosen to compare the toxicity effects of AgNPs and silver ions. The toxicity of AgNPs has been extensively compared with silver ion exposure alone. A summary of the current knowledge on toxicity impacts of AgNPs and silver ion is shown in Table 1. The literature reports (Table 1) that exposure to either AgNPs or silver ions alone may induce similar phenotypic abnormalities, such as formation of oedema [43], slower swim bladder inflation [20], decreased ATPase enzyme activity [23] and altered expression of similar genes associated with oxidative phosphorylation, detoxifying processes and protein synthesis [18,22,44]. In terms of contrasting impacts between AgNPs and silver ions alone, exposure to AgNPs alters the gene expression of metal-sensitive metallothioneins [22], while no such silver ion induction has been reported to date. AgNP exposure also induces delayed hatching [43], yet silver ions accelerate hatching [45]. It is evident that there may be some shared toxicity mechanisms between AgNPs and silver ions, while other mechanisms may be unique. In this study, silver ion exposure alone did not induce the same oxidative stress or *Pxmp2* response at the concentrations studied, suggesting that the molecular responses were specific to the nanoparticles rather than to any disassociated metal ion elements. The nanomaterial coating may also be a confounding factor in toxicity assessments [20,24,30,46], though oleic acid was used in the present study and is regarded as non-toxic [47]. AgNPs coated with fatty acid (oleic acid), used in the present study, are quite stable and do not dissolute as much silver ion as previously reported [22,48]. The limited release of silver ion from the AgNPs used might explain the observed limited toxicity in fish embryos.

## 4. Materials and Methods

### 4.1. Materials and Characterisation of AgNPs

AgNPs were purchased in powder form from Suzhou Cold Stones Technology Co., Ltd. (Suzhou Cold Stones Technology Co. Ltd., Suzhou, China). AgNPs had an approximate diameter of 4 or 10 nm, designated as S4 and S10 AgNPs, and were coated with 10%–13% fatty acid (oleic acid). AgNO_3_ (CAS 7761–88-8, 99% purity) used for the gene expression control group was supplied by Sigma-Aldrich (St. Louis, MO, USA).

The properties of AgNPs were characterised by transmission electron microscopy (TEM), and TEM images were taken using a JEOL JEM-2100 TEM microscope (Jeol Ltd., Tokyo, Japan). AgNPs were dispersed in deionised water and sonicated at 40 kHz for 20 min. A small drop of the suspension was placed on a copper grid and air-dried at room temperature. The TEM images were used to provide information on the structure, size, size distribution and morphology of the S4 and S10 range AgNPs.

### 4.2. Zebrafish Maintenance and Embryo Collection

Wild-type adult zebrafish were kept in a laboratory fish tank at 28 °C with a cycle of 14:10 h of light:dark, in a closed flow-through system with charcoal-filtered tap water [49]. The fish were fed twice a day with dry flake feed and brine shrimp. Before egg collection, female and male fish were placed in a clean breeding tank at a sex ratio of 1:2. Spawning was induced in the morning when the lights came on after the 10 h dark period. Embryos were then collected 0.5 h later and rinsed with system water. Collected eggs were washed with clean water and then screened under a dissection microscope (SMZ168, Motic, Xiamen, China). The developmental stage of embryos was identified according to Kimmel et al. [50].

### 4.3. Exposure Design

Healthy fish embryos were selected and transferred at 4 hpf into 12 well cell culture plates containing 3 mL of fish culture solution in each well. Experiments were conducted, and ethical approval sought, in accordance with the rules and guidelines of the institution and country. The dispersed S4 AgNPs were prepared at concentrations of 0.481, 0.963 and 1.925 mg/L. The dispersed S10 AgNPs were also prepared at concentrations of 0.481, 0.963 and 1.925 mg/L. All treatments were performed in quadruplicate with 40 embryos in each replicate, and 160 embryos exposed in total for each dose. Treatment lasted from 4 hpf to 96 hpf in a dark environment without media replacement. Two independent exposure experiments were prepared: one for the morphological observation and ICP-MS analysis, and the other for the mRNA expression analysis. For the control treatment groups, one control comprised medium only (‘control’); for the gene expression analysis only, there was a second control treatment group containing silver ions as AgNO_3_ (0.018 mg/L of Ag+) (‘AgNO_3_ control’). Similarly, the control treatment groups comprised four wells, each containing 40 individual embryos.

In the morphological observation and ICP-MS analysis, 40 embryos from each concentration (10 embryos per replicate and four replicates per treatment) were randomly selected for photography. The embryos were anaesthetised with 0.024% tricaine (Sigma-Aldrich) at 72 hpf. The body length and yolk sac area of the embryos were examined under a Zeiss stereomicroscope (V8, Göttingen, Germany), equipped with an AxiocamICc3 photomicrography system. After the measurement, the larvae were returned to the well for the 96 hpf exposure period ICP-MS analysis. In the mRNA expression analysis, embryos were stored at 96 hpf in RNAlater^®^ solution at −80 °C until quantitative (Q) PCR analysis.

### 4.4. Uptake of AgNPs in Fish Embryos 

The uptake of AgNPs was studied by measuring the silver content in the exposed zebrafish embryos at 96 hpf. As a pre-treatment, to remove surface AgNPs on the embryos, 40 embryos in each replicate were washed three times with 1× phosphate-buffered saline. For digestion, embryos were dissolved with 2 mL of concentrated HNO_3_ for 2 h at 180 °C; 1 mL of 30% H_2_O_2_ was added for the following 1 h at 120 °C. The samples were then diluted to a final concentration of 2% HNO_3_ with deionised water. The pre-treatment of the samples was carried out according to previous reports [22,51] to determine the silver content using inductively coupled plasma-mass spectrometry (ICP-MS; Neptune; Thermo Fisher Scientific, MA, USA). The wet weight of each replicate was measured, and silver content was calculated via (total silver/wet weight) for each replicate. The background silver content in the tissues of control fish was subtracted from that of the treated samples. The charcoal-filtered tap water used to breed the parental adult zebrafish contained a small amount of silver [52,53]. A total of 160 embryos for each concentration (40 embryos per replicate and four replicates per treatment) were used for ICP-MS measurement.

### 4.5. Target Gene Isolation and Characterisation

Total RNA was extracted from embryos exposed to the S4 and S10 AgNPs and treatment control AgNO_3_ collected at 96 hpf, as well as the corresponding control treatment group, using the manufacturers protocol (Roche Diagnostics GmbH, Mannheim, Germany). To assess the integrity of total RNA, samples were analysed on a denaturing agarose gel stained with ethidium bromide (Invitrogen™, Waltham, MA, USA). cDNA was prepared using SuperScript VILO cDNA synthesis reagents and its protocol (Life Technologies, Paisley, U.K.), with 14 μL (100 ng) of total RNA. In a 0.2 mL tube, the following reagents were added: 4 μL of 5× VILO^TM^ Reaction Mix (includes random primers, MgCl_2_, and dNTPs in a buffer formulation) and 2 μL of 10X Superscript enzyme mix. Each reaction was incubated at 25 °C for 10 min, and then 60 min at 42 °C, followed by 5 min at 85 °C and a holding step at 4 °C. To degrade any remaining RNA, the following reagents were added: 0.5 μL (5 units) of RNase H (supplied in 100 mM KCl, 20 mM Tris-HCl (pH 7.5), 10 mM MgCl_2_, 0.1 mM EDTA, 0.1 mM dithiothreitol and 50% glycerol) and 2 μL of 10X RNase H Reaction Buffer (includes 75 mM KCl, 50 mM Tris-HCl, 3 mM MgCl_2_, 10 mM MgCl_2_ in pH 8.3 at 25 °C). All reagents were mixed, incubated at 37 °C for 45 min and then stored at −20 °C.

For the generation of *SOD2*, *CAT, HIF*, *Pxmp2* and *Muc* PCR products, 1 μL of cDNA was combined with 0.5 μL of 10 mM dNTPs, 5 μL of amplification buffer, 0.5 μL of 0.5–4.5 mM MgCl_2_, 0.5 μL of 1.5 μM of each sense and antisense primers (Table 2) and 0.25 μL (1.25 units) of Herculase II fusion DNA polymerase (Agilent Technologies, Santa Clara, CA, U.S.A.) for a total reaction volume of 25 μL. *Elongation factor 1* (*EF1*), *18S rRNA* (*18S*) and *β tubulin* were evaluated as potential reference genes. Amplifications were carried out using the TC-4000 Thermal Cycler (Techne, Staffordshire, U.K.) equipped with a heated lid. All reactions were initially denatured at 94 °C for 30 s, then cycled 35 times with 30 s denaturation at 94 °C, 30 s annealing at 50/55/60 °C and 30 s elongation at 72 °C. Finally, a 2 min extension step was conducted at 72 °C. The PCR fragments were sequenced commercially by Macrogen (Amsterdam, Netherlands). Identities of PCR fragments were verified using a blast search on the NCBI database (http://blast.ncbi.nlm.nih.gov/Blast.cgi), and aligned using a multiple sequence alignment program, Clustal Omega (http://www.ebi.ac.uk/Tools/msa/clustalo/).

### 4.6. Quantitative (Q) PCR Analysis of mRNA Expression

qPCR was carried out using 20 μL reaction volumes consisting of 10 μL of SYBR^®^ Green Master Mix (Roche, U.K.), 7 µL of sterilised water, 1 µL of the cDNA template and 2 µL of optimised primer concentration (*EF1*, *CAT*, *HIF*: 200 nM; *18S*, *Pxmp2, Muc*: 300 nM, *SOD2*: 400 nM). Two reference genes (*EF1* and *18S*) were determined to be the most stable across treatment groups using geNorm software. 

Amplifications were carried out using the CFX96™ Real-Time PCR system, C1000™ Thermal Cycler (Bio Rad, Watford U.K.), in duplicate and with negative controls. Reactions started with denaturation at 50 °C for 2 min, 95 °C for 10 min, followed by 40 cycles of a three-step protocol starting with denaturation at 95 °C for 10 s, followed by annealing at 60 °C for 1 min, then 72 °C for 1 min. At the end, a melting/dissociation curve was established. A relative quantification method was used to determine changes in target gene expression in the treatment group compared to untreated control samples, using the geomean of the reference genes for normalisation and the ΔCt method [54].

### 4.7. Statistical Analyses 

The particle size of the AgNPs, the zebrafish larvae body length and yolk sac area were all measured and calculated using ImageJ2x (National Institutes of Health, Bethesda, MD, USA) and GraphPad Prism 5 (GraphPad Software, La Jolla, CA, USA). Significant difference in body length/yolk sac area was determined using single-way analysis of variance and Dunnett’s post hoc test. The analysis of variance was performed with GraphPad Prism 5 (*p* < 0.05). Significant difference in relative gene expression was tested using either a two-sample *t*-test or Mann–Whitney U test (SPSS, Version 22, IBM Corp. New York, NY, USA).

## 5. Conclusions

In summary, we studied the comparative uptake and developmental toxicity of 4 and 10 nm size range AgNPs in zebrafish embryos. Under the exposure conditions used in this study, S4 AgNPs at the higher dose level (of 1.925 mg/L) were taken up by zebrafish embryos. The uptake was correspondingly associated with significant developmental toxicity (delayed development). Transcription of two genes related to hypoxia and membrane transport was impacted by S4 AgNPs but not S10 AgNPs. A stable surface coating may help to limit the release of silver ion and associated toxicity of studied AgNPs in fish embryos, and particle size may also act as another possible factor in determining the uptake and developmental toxicity of AgNPs in fish embryos.

## Figures and Tables

**Table 1:** A summary of the observed toxicity impacts of AgNPs and silver ions reported in the literature.

Organism	Source	Exposure Period	NP Sizes	Exposure Concentration	Ag Uptake of Negative Control	Ag Uptake	Biological Effects	References
**Zebrafish** **Embryos**	AgNPs	0–4 days	3–8 nm	0.1 and 0.125 mg/L	—	—	Induced delayed hatching and formation of oedema.	[43]
	AgNO_3_		—	0.04 and 0.08 mg/L			Ag^+^ decreased absorption of yolk sac and resulted in pericardial oedema.	
**Zebrafish** **Embryos**	AgNPs	6–120 hpf	10 nm	1.7 and 5.1 mg/L	—	—	AgNPs induced slowed swim bladder inflation.	[20]
	AgNO_3_		—	0.51, 1.7 and 5.1 mg/L			Ag^+^ slowed swim bladder inflation.	
**Zebrafish** **Embryos**	AgNPs	4–24 and 4–48 hpf	10 nm	0.005 mg/L	—	—	AgNPs altered gene expression associated with oxidative phosphorylation and protein synthesis.	[44]
	AgNO_3_	4–24 hpf	—	0.00025 mg/L			Ag^+^ altered oxidative phosphorylation and protein synthesis gene expression.	
**Adult** **Zebrafish**	AgNPs	0–4 days	20 nm	1 mg/L	Gill and intestine: No uptake detected	Gill: 5.11 μg/adult Intestine: 0.68 μg/adult	AgNPs decreased ATPase enzyme activity.	[23]
	AgNO_3_		—			Gill: 1.66 μg/adult Intestine: 2.29 μg/adult	Ag^+^ decreased ATPase enzyme activity.	
**Zebrafish** **Embryos**	AgNPs	4–96 hpf	10 nm	1.925 mg/L	Head and trunk: No uptake detected	Head: 0.03 ng/embryo Trunk: 0.05 ng/embryo	AgNPs induced small head, small eye and cardiac defects, and altered the gene expression of neural development-related, metal-sensitive metallothioneins, and detoxification.	[22]
**Zebrafish** **Embryos**	AgNPs	4–96 hpf	4 nm	1.925 mg/L	Whole body tissue: no uptake detected	Whole body tissue: 21.47 ng/g	AgNPs decreased body length, delayed yolk sac absorption, and altered oxidative stress and channel-forming protein gene expression.	This study
**Adult** **Zebrafish**	AgNPs	0–48 h	26.6 ± 8.8 nm	1 mg/L	Carcass: 0.02 ng/g	Carcass: 0.47 ng/g	AgNPs altered signal transducer activity, enzyme activity and membrane function gene expression.	[21]
**Zebrafis** **Embryos**	AgNO_3_	1–24, 1–48 or 1–96 hpf	—	0.02 mg/L	—	—	Ag^+^ altered detoxifying processes and oxidative stress gene expression.	[44]
**Whitefis** **Embryos**	AgNO_3_	hatch	—	0.1 mg/L	—	—	Ag^+^ accelerated hatching and decreased absorption of yolk sac.	[46]

**Table 2:** Primer pairs used for the isolation of candidate and reference genes from zebrafish embryos.

Targeted Transcript	Forward Primer (5′-3′)	Reverse Primer (5′-3′)	Amplicon Size (bp)
*18S*	TAGAGGGACAAGTGGCGTTC	CCTCGTTGATGGGAAACAGT	195
*EF1*	GATGCACCACGAGTCTCTGA	TGATGACCTGAGCGTTGAAG	158
*MUC*	TGGGATCGCAAAACCACTGT	GTTGTGCATCAGGGCAAGTG	187
*PXMP2*	AGTTACTGGCCTGCGATGAA	CACTGCAGTAAGGCACAACC	157
*HIF*	AGACTGCCACGGAAAAGCTA	CCAGAGGCAGAAGAGCAGTT	161
*CAT*	GCGGATACCAGAGAGAGTCG	ATCTGATGACCCAGCCTCAC	172
*SOD2*	AGCGTGACTTTGGCTCATTT	ATGAGACCTGTGGTCCCTTG	166
